# Variables Associated with Knowledge of the Fertile Window in Women and Men in Colombia and Ecuador

**DOI:** 10.1177/26884844251374979

**Published:** 2025-09-15

**Authors:** A. Rodríguez-López, M.I. Griffin-Valdivieso, A.C. Marí-Espejo

**Affiliations:** ^1^ Latin American Institute of the Family (ILFARUS), Universidad de la Sabana, Chía, Colombia.; ^2^ ILFARUS, Universidad de la Sabana, Chía, Colombia.

**Keywords:** fertility awareness, fertile window, reproductive health, natural family planning

## Abstract

**Purpose:**

Accurate knowledge of the fertile window—the period within the ovulatory cycle when conception is most likely—is important to reproductive health and informed family planning. Prior research has consistently documented low to moderate awareness of this concept, particularly among young adults. This study examined sociodemographic and reproductive variables associated with fertile window knowledge among individuals of reproductive age in Colombia and Ecuador.

**Methods:**

Data were drawn from a cross-sectional analysis within the ongoing international “FERTIPLAN” project, which investigates fertility intentions in Latin America. The sample comprises 1456 participants (73.4% women, 26.6% men) aged 18–44 years from Colombia and Ecuador.

**Results:**

Women were nearly twice as likely as men to demonstrate knowledge of the fertile window (odds ratio [OR] = 1.98; 95% confidence interval [CI]: 1.32–2.97). All age groups aged 25 years and older exhibited significantly higher odds of fertile window knowledge compared with the 18–24 age group (ORs ranging from 1.89 to 2.45), but no significant differences were observed among the older age groups, indicating that the primary knowledge gap lies between the youngest cohort and all older age groups. Participants with higher income (OR = 2.59; 95% CI: 1.54–4.35), fertility intentions (OR = 1.47; 95% CI: 1.06–2.04), and familiarity with fertility awareness-based methods (OR = 2.55; 95% CI: 1.58–4.12) had significantly greater knowledge of the fertile window.

**Conclusions:**

These findings underscore the influence of sex, age cohort, socioeconomic status, and reproductive factors on fertility awareness, offering valuable insights for advancing preconception care and addressing knowledge gaps in Latin America.

## Introduction

### Background

The fertile window, defined as the 6-day interval encompassing the 5 days preceding ovulation and the day of ovulation itself, represents the period of peak fecundability within the menstrual cycle.^1,2^ This window is determined by the survival of sperm in the female reproductive tract for up to 5 days and the viability of the ovum for approximately 24 hours postovulation.^3^ Although menstrual cycle length varies, ovulation generally occurs around the midpoint, making this the most favorable time for conception.^4,5^

Knowledge of the fertile window enables women to optimize timing for pregnancy achievement or avoidance, while also facilitating early detection of reproductive health issues.^3,6–9^ Moreover, fertility awareness empowers individuals to develop a deeper understanding of their bodies and natural reproductive processes, potentially fostering healthier attitudes and behaviors regarding sexuality and emotional and relational well-being.^6^ Conversely, a lack of knowledge can contribute to unintended pregnancies, spontaneous or induced abortions, and undiagnosed infertility^7,10^

Global research has consistently highlighted low levels of fertile window awareness across diverse populations. Studies in the United States,^11–13^ several African countries,^10,14–17^ New Zealand,^18^ and Haiti^7^ report that even educated groups, such as university students and health care professionals,^19^ struggle to accurately identify this phase. Similarly, research assessing fertility awareness among women attending fertility clinics in Australia^8^ and India^20^ has indicated that few women can accurately identify the most fertile phase of the menstrual cycle.

In Latin America, however, data on fertility awareness remain limited, despite the region’s unique sociocultural and health care contexts. This gap is particularly concerning given the rising interest in fertility awareness-based methods (FABMs), driven by concerns over hormonal contraceptives’ side effects, including mood alterations, weight gain, hypertension, cardiovascular risks, and potential oncogenic effects.^16,21^

Sociodemographic variables, including age, educational attainment, and socioeconomic status, have been identified as predictors of fertility awareness. Older individuals, those with higher education levels, and those from higher-income backgrounds tend to have a more thorough understanding of the fertile window.^10,13,16,17,22,23^

Deficiencies in sexual and reproductive education often exacerbate awareness gaps. Many educational programs focus primarily on preventing sexually transmitted infections (STIs) and unexpected pregnancies, sidelining fertility health and infertility prevention.^12,20,24^ A more holistic approach, integrating fertility awareness, could enhance family planning, reduce unplanned pregnancies, and promote earlier identification of reproductive health issues.^19^

### Objectives

Given the persistent low levels of fertility awareness worldwide, increasing dissatisfaction with hormonal contraceptives,^16,25^ and rising infertility rates coupled with prolonged time-to-conception,^23^ evaluating knowledge of the fertile window in Latin America remains a relevant area of investigation. In light of the limited research on this topic in the region, this study aimed to evaluate the sociodemographic and reproductive variables associated with knowledge of the fertile window among women and men in Colombia and Ecuador. By addressing this regional research gap, the findings may contribute to the development of evidence-based preconception health strategies and support efforts to enhance fertility awareness across diverse populations in Latin America.

## Materials and Methods

### Design and sample

The data were collected between September 2023 and September 2024 through the “FERTIPLAN” project on fertility and family planning at the Latin American Institute of the Family (ILFARUS). For this study, 15 questions were selected from the 118 questions included in the “FERTIPLAN” project to address the research question. Data collection was conducted *via* an anonymous, self-administered online questionnaire in Spanish. A non-probability convenience sampling method was employed for participant selection. The FERTIPLAN questionnaire was distributed through the social media platforms of public and private educational and sociocultural institutions from various cities and countries in Latin America. Potential participants were invited to complete the online survey and encouraged to share it with contacts who met the inclusion criteria. The study was presented as an anonymous survey on fertility and family planning, and participation was entirely voluntary.

The inclusion criteria were (1) being between 18 and 44 years of age and (2) having Colombian or Ecuadorian nationality. The final sample comprised 1456 participants, including 1069 women (78.5% from Colombia and 21.5% from Ecuador) and 387 men (81.9% from Colombia and 18.1% from Ecuador). The study protocol was approved by the ILFARUS research subcommittee prior to data collection and adhered to the principles outlined in the Declaration of Helsinki. Participants were informed about the study’s purpose and provided their consent at the beginning of the virtual survey for voluntary participation and data usage for research purposes.

### Study measures

#### Dependent variable: fertile window knowledge

Although no standardized methodology exists for assessing knowledge of the fertile window, this study adapted four items based on fertility biomarkers, the menstrual cycle’s fertile period, and sperm survival in the vaginal tract.^7,8,10,16,22,26^

Participants answered the following four questions:

• “Can women identify the fertile days of their menstrual cycle by recognizing ovulation through variations in vaginal discharge?”. (Correct answer: True).
• “Can sperm survive for up to 5 days inside the vaginal tract?” (Correct answer: True).
• “Can women get pregnant on any day of the menstrual cycle?” (Correct answer: False).
• “When is the most fertile time in a woman’s menstrual cycle?” with response options: (a) During her menstruation, (b) After menstruation ends, (c) In the middle of the cycle, (d) Before menstruation begins, (e) At any time during the cycle, (f) Other (Correct answer: c).

The first three questions were presented in a true/false format, with correct answers (true for questions 1 and 2; false for question 3) coded as 1 and incorrect answers coded as 0, consistent with established evidence.^3,6,26,27^ The fourth question, a multiple-choice item, had the correct answer (c) coded as 1, while incorrect responses (a, b, d, e, f) were coded as 0, based on menstrual cycle literature.^3^

#### Independent variables

Sociodemographic variables analyzed included age (categorized into five groups: 18–24, 25–29, 30–34, 35–39, and 40–44 years), nationality (Colombian or Ecuadorian), marital status (single, in a relationship or dating, married, de facto marital union, separated/divorced, widowed), educational level (categorized in “primary and secondary”; “technical and university”; “postgraduate”), and socioeconomic status (low, medium, or high).

Religiosity was measured using the question: “How often do you practice your religious activity (going to church or temple, praying or doing prayer, religious rituals, *etc.*)?” with response options from 0 (never) to 5 (more than once a week).^28^ This variable was dichotomized into “high religiosity” (attending once a week or more) and “null/low religiosity” (never, almost never, a few times a year, and a few times a month).

Sexual and reproductive health variables were assessed through five questions. The following factors were considered:

• Number of children (0, 1, 2, or 3 or more).
• Fertility intention (“Would you like to have children or more if you already have them?” with “yes” or “no” responses).
• Use of family planning methods (categorized as 0 “no planning,” 1 “use of FABMs,” and 2 “use of artificial methods” such as condoms, hormonal, or surgical methods).
• Knowledge of FABMs (“None,” “I have heard something,” “I have received an informative talk,” “I have read a specific book,” “I have been taught by a specialized instructor”).
• Intention to use FABMs (dichotomized as “yes” or “no”).

#### Data analysis

To define the dependent variable, “*knowledge of the fertile window*,” we constructed an index using four items from the FERTIPLAN questionnaire. Based on international literature, the item “*When is the most fertile time in a woman’s menstrual cycle?*” emerged as the strongest predictor of fertile window knowledge. Therefore, we applied a differential weighting scheme: this item was assigned a weight of 1.4, while the remaining three items—“*recognition of ovulation by cervical mucus,*” “*sperm survival up to 5 days,*” and “*pregnancy possible on any day*”—were each assigned a weight of 1.2. This approach is grounded in international evidence and aligns with methodological recommendations.^6,7,10,11^

The index was calculated as the weighted sum of correct responses (maximum score = 5). A cutoff score of 3.8 was established to classify “*adequate knowledge*,” ensuring that only participants who correctly identified the most fertile moment in the cycle were considered knowledgeable. This multidimensional approach captures not only *when* the fertile window occurs but also *how* and *why*, thus reducing random error and yielding a more robust measure.

Internal consistency was evaluated using Kendall’s Tau-b correlation matrix, appropriate for short scales with dichotomous or ordinal items.^29^ All pairwise correlations were positive and statistically significant (range: 0.027–0.162; all *p* < 0.01), supporting the index’s unidimensionality and construct validity. The complete correlation matrix is presented in Table 1 of the Results section.

**Table 1.** Kendall’s Tau-b Correlation Matrix Among Items of the Fertile Window Knowledge Index

Item	Item 1	Item 2	Item 3	Item 4
Ovulation recognition by cervical mucus	—			
Sperm survival	0.162***	—		
Pregnancy on any day of the cycle	0.082**	0.027	—	
Most fertile moment of the cycle	0.125***	0.070**	0.116***	—

Note: All Kendall’s Tau-b coefficients are positive and statistically significant (*p* < 0.05, ** *p* < 0.01, *** *p* < 0.001).

Univariate analyses (frequencies and percentages) were conducted to describe the main demographic and reproductive characteristics of the sample by nationality and the general population knowledge of the fertile window. Differences between sociodemographic and reproductive variables concerning fertile window knowledge were examined using chi-square (χ^2^) test. Statistical significance was established at a *p-*value <0.05.

A binomial logistic regression model was used to identify predictors of fertile window knowledge, controlling for sociodemographic, health, and reproductive variables. Odds ratios (ORs) and 95% confidence intervals (CIs) were estimated.

Data were collected using Microsoft Forms and analyzed with Jamovi 2.3.28 statistical software.

## Results

### Construction and validation of a fertile window knowledge index

The internal consistency of the fertile window knowledge index was assessed using Kendall’s Tau-b correlation matrix (Table 1). All inter-item correlations were positive and statistically significant, supporting the unidimensional structure of the index and the consolidation of the four items into a single, coherent measure of fertile window knowledge.

### Sociodemographic and reproductive characteristics

Table 2 summarizes the main sociodemographic and reproductive characteristics of the sample (*N* = 1,456). The majority of participants were women (73.4%) and aged 18–34 years (72.1%). Approximately half had a technical or university education (51.7%) and were single (46.2%). Regarding reproductive factors, 70.1% of the participants had no children, although 56.3% expressed the intention to have children in the future (Table 2).

**Table 2.** Descriptive Analysis of Sociodemographic and Reproductive Factors of the Sample (*n* = 1.456 Men and Women)

Variable	Women	Men	Total
	1069 (73.4%)	387 (26.6%)	1456 (100%)
Nationality			
Colombia	839 (78.5)	317 (81.9)	1156 (79.4)
Ecuador	230 (21.5)	70 (18.1)	387 (20.6)
Age			
18–24	363 (34.0)	192 (33.3)	555 (38.1)
25–29	170 (15.9)	68 (17.6)	238 (16.3)
30–34	203 (19.0)	54 (14.0)	257 (17.7)
35–39	170 (15.9)	27 (7.0)	197 (13.5)
40–44	163 (15.2)	46 (11.9)	209 (14.4)
Marital status			
Single	448 (41.9)	224 (57.9)	672 (46.2)
Dating	215 (20.1)	89 (23.0)	304 (20.9)
Married	283 (26.5)	54 (14.0)	337 (23.1)
Cohabitation	100 (1.3)	14 (3.6)	114 (7.8)
Separated/divorced	23 (9.4)	6 (1.6)	29 (2.0)
Educational level			
Primary and high school	188 (17.6)	110 (28.4)	298 (20.5)
Technical and university	548 (51.3)	205 (53.0)	753 (51.7)
Postgraduate	333 (31.1)	72 (18.6)	405 (27.8)
Socioeconomic status			
Low	310 (29.0)	154 (39.8)	464 (31.9)
Middle	603 (56.4)	190 (49.1)	793 (54.5)
High	156 (14.6)	43 (11.1)	199 (13.7)
Religiosity			
Non-religious	624 (58.4)	278 (71.8)	902 (62.0)
Religious	445 (41.6)	109 (28.2)	554 (38.0)
Fertility intentions^a^			
No	492 (46.0)	144 (37.2)	636 (43.7)
Yes	577 (54.0)	243 (62.8)	820 (56.3)
Have children			
No children	697 (65.2)	323 (83.5)	1020 (70.1)
One child	172 (16.1)	29 (7.5)	201 (13.8)
Two children	129 (12.1)	24 (6.2)	153 (10.5)
Three or more children	71 (6.6)	11 (2.8)	82 (5.6)
Knowledge of FABMs^b^			
None	316 (29.6)	158 (40.8)	474 (32.6)
I have heard something	292 (27.3)	100 (25.8)	392 (26.9)
I have received some informative talks	262 (24.5)	72 (18.6)	334 (22.9)
I have read a specific book	54 (5.1)	22 (5.7)	76 (5.2)
I have been taught by a specialized instructor	145 (13.6)	35 (9.0)	180 (12.4)
Intention to use FABMs^b^			
No	646 (60.4)	211 (54.5)	857 (58.9)
Yes	423 (39.6)	176 (45.5)	599 (41.1)
Family planning methods			
FABMs^b^	81 (7.6)	14 (3.6)	95 (6.5)
Artificial methods	504 (47.1)	170 (43.9)	674 (46.3)
They do not plan	484 (45.3)	203 (52.5)	687 (47.2)
Knowledge of the fertile window			
Know	204 (19.1)	35 (9.0)	239 (19.4)
Not know	865 (80.9)	352 (91.0)	1217 (83.6)

^a^ Intending to have a child.

^b^ Fertility awareness-based methods (FABMs).

### Knowledge of the fertile window in women and men according to sociodemographic and reproductive variables

Descriptive analysis revealed low knowledge of the fertile window among women (19.1%) and men (9%). Bivariate analysis showed statistically significant differences in fertile window knowledge between women and men in Colombia and Ecuador (Table 3). Among women, significant associations (*p* < 0.05) were found with age, marital status, educational level, socioeconomic level, and religiosity. Regarding reproductive variables, significant associations (*p* > 0.01) were observed for fertility intentions, having children, knowledge of FABMs, and intention to use FABMs. In men, these associations were less consistent and significant overall (Table 3).

**Table 3.** Chi-Square Association Between Sociodemographic and Reproductive Variables and Knowledge of the Fertile Window

	Women			Men		
Variable	Known fertile window (%)	No knowledge fertile window (%)	*p*-Value	Known fertile window (%)	No knowledge fertile window (%)	*p-*Value
Nationality						
Colombia	160 (19.1)	679 (80.9)	0.98	27 (8.5)	290 (91.5)	0.44
Ecuador	44 (19.1)	186 (80.9)		8 (11.4)	62 (88.6)	
Age						
18–24	30 (8.3)	333 (91.7)	<0.001^*^	8 (4.2)	184 (95.8)	0.01
25–29	34 (20.0)	136 (80.0)		10 (14.7)	58 (85.3)	
30–34	46 (22.7)	157 (77.3)		5 (9.3)	49 (90.7)	
35–39	48 (28.2)	122 (71.8)		6 (21.4)	22 (78.6)	
40–44	46 (28.2)	117 (71.8)		7 (15.2)	39 (84.8)	
Marital status						
Single	61 (13.6)	387 (86.4)	<0.001^*^	19 (8.5)	205 (91.5)	0.274
Dating	36 (16.7)	179 (83.3)		6 (6.7)	83 (93.3)	
Married	89 (31.4)	194 (68.6)		9 (16.7)	45 (83.3)	
Cohabitation	11 (11.0)	89 (89.0)		1 (7.1)	13 (92.9)	
Separated/divorced	7 (30.4)	16 (69.6)		0 (0.0)	6 (100)	
Educational level						
Primary and high school	14 (7.4)	174 (92.6)	<0.001^*^	3 (2.7)	107 (97.3)	0.002
Technical and university	89 (16.2)	459 (83.8)		19 (9.3)	186 (90.7)	
Postgraduate	101 (30.3)	232 (69.7)		13 (18.1)	59 (81.9)	
Socioeconomic status						
Low	26 (8.4)	284 (91.6)	<0.001^*^	10 (6.5)	144 (93.5)	0.35
Middle	124 (20.6)	479 (79.4)		20 (10.5)	170 (89.5)	
High	54 (34.6)	102 (65.4)		5 (11.6)	38 (91)	
Religiosity						
Non-religious	91 (14.6)	533 (85.4)	<0.001^*^	260 (93.5)	18 (6.5)	0.005
Religious	113 (25.4)	332 (74.6)		92 (84.4)	17 (15.6)	
Fertility intention						
No	73 (14.8)	419 (85.2)	0.001^*^	13 (9.0)	131 (91.0)	0.99
Yes	131 (22.7)	446 (77.3)		22 (9.1)	221 (90.9)	
Have children						
No children	111 (15.9)	586 (84.1)	<0.001^*^	25 (7.7)	298 (92.3)	0.23
One child	38 (22.1)	134 (77.9)		4 (13.8)	25 (86.2)	
Two children	30 (23.3)	99 (76.7)		4 (16.7)	20 (83.3)	
Three or more children	25 (35.2)	46 (64.8)		2 (18.2)	9 (81.8)	
Knowledge of FABMs^**^						
None	34 (10.8)	282 (89.2)	<0.001^*^	11 (7.0)	147 (93.0)	0.084
I have heard something	47 (16.1)	245 (83.9)		5 (5.0)	95 (95.0)	
I have received some informative talks	53 (20.2)	20 (79.8)		10 (13.9)	62 (86.1)	
I have read a specific book	16 (29.6)	38 (70.4)		3 (13.6)	19 (86.4)	
I have been taught by a specialized instructor	54 (37.2)	91 (62.8)		6 (17.1)	29 (82.9)	
Intention to use FABMs^**^						
No	73 (11.3)	573 (88.7)	<0.001^*^	18 (8.5)	193 (91.5)	0.70
Yes	131 (31.0)	292 (69.0)		17 (9.7)	159 (90.3)	

^*^ Intending to have a child.

^**^ Fertility awareness-based methods (FABMs).

Notably, married women had the highest level of knowledge (31.4%) compared with single women (13.6%) (*p* < 0.001). Likewise, women from high socioeconomic background exhibited greater knowledge (34.6%) compared with those from a low socioeconomic background (8.4%) (*p* < 0.001). This trend was also observed in fertility intentions, having children, and knowledge of FABMs. No significant differences were found by nationality (Table 3).

### Multivariate analysis

Multivariate analysis (Table 4) indicated that women were twice as likely as men to have knowledge of the fertile window (OR = 1.98; 95% CI: 1.32–2.97). All age groups aged 25 and older exhibited significantly higher odds of fertile window knowledge compared with the 18–24 age group. Specifically, the ORs for the 25–29, 30–34, 35–39, and 40–44 age groups were 2.44 (95% CI: 1.45–4.09), 1.89 (95% CI: 1.11–3.21), 2.08 (95% CI: 1.14–3.81), and 2.45 (95% CI: 1.40–4.29), respectively, relative to the youngest cohort (Table 4). No statistically significant differences were observed among the 25–44 age groups, indicating that the primary knowledge gap lies between the 18–24 cohort and all older age groups.

**Table 4.** Sociodemographic and Sexual and Reproductive Health Variables Associated with Knowledge of the Fertile Window

Variables	*p*-Value	OR [95% CI]
Sex:		
Woman–men	<0.001	1.98 [1.32–2.97]^***^
Age:		
25 to 29–18 to 24	<0.001	2.44 [1.45–4.09]^***^
30 to 34–18 to 24	0.019	1.89 [1.11–3.21]^*^
35 to 39–18 to 24	0.002	2.40 [1.38–4.16]^**^
40 to 44–18 to 24	0.002	2.45 [1.40–4.29]^**^
Socioeconomic status:		
Middle–low	0.031	1.58 [1.04–2.40]^*^
High–low	<0.001	2.59 [1.54–4.35]^***^
Educational_level:		
Technical and university–elementary and high school	0.123	1.59 [0.88–2.86]
Postgraduate–elementary and high school	0.037	2.01 [1.04–3.88]^*^
Fertility intention:		
Wants children–does not want children	0.020	1.47 [1.06–2.04]^*^
Know FABMs:		
I have heard something—none	0.669	1.10 [0.70–1.72]
I have received lecture—none	0.103	1.45 [0.92–2.27]
Read a specific book—none Instruction by a specialized instructor—none	0.034	2.04 [1.05–3.94]^*^
Intention to use_FABMs:	<0.001	2.55 [1.58–4.12]^***^
Yes–no	<0.001	1.82 [1.31–2.52]^***^

This association was adjusted for other variables such as nationality, marital status, religiosity, childbearing, current planning method, with no statistically significant associations.

^*^ *p < 0.05, **p < 0.01, ***p < 0.001.

CI, confidence interval; FABMs, Fertility awareness-based methods; OR, odds ratio.

Participants from low socioeconomic backgrounds were less likely to have fertile window knowledge (OR = 2.59; 95% CI: 1.54–4.35) compared with those from high socioeconomic backgrounds. Those with fertility intentions were 1.47 times more likely to have this knowledge (OR = 1.47; 95% CI: 1.06–2.04). Participants who received instruction on FABMs from a specialist were 2.55 times more likely to have knowledge of the fertile window (OR = 2.55; 95% CI: 1.58–4.12). Similarly, those intending to use family planning methods were 1.82 times more likely to have this knowledge (OR = 1.82; 95% CI: 1.31–2.52) (Table 4).

The results indicate that knowledge about the fertile window is significantly associated with sex, age, socioeconomic status, fertility intentions, and knowledge and intention to use FABMs.

## Discussion

This study aimed to evaluate the variables associated with knowledge of the fertile window among women and men of reproductive age in Colombia and Ecuador, with the goal of informing targeted reproductive health education strategies. Among the 1456 participants analyzed, only 16.4% demonstrated adequate knowledge of the fertile window, a proportion consistent with findings from international studies across diverse settings, including the United States, African nations, New Zealand, Haiti, Australia, and India.^7,8,10–18,20^ The observed lack of knowledge may be partly attributable to deficiencies in comprehensive educational programs addressing the ovulatory cycle and fertile window within public health curricula, as suggested by prior research.^6,20,24,26^ In addition, limited access to reliable, culturally tailored reproductive health information may exacerbate this issue, particularly in Latin America, where research on fertility awareness remains scarce.

Sociodemographic variables, including sex, age, and socioeconomic status, emerged as significant predictors of fertility window knowledge. Women were twice as likely as men to possess accurate knowledge, a disparity consistent with literature highlighting the frequent exclusion of men from reproductive health education initiatives literature.^30–32^ Similarly, participants of higher socioeconomic status demonstrated significantly greater awareness than those of lower status, reflecting the role of economic resources in facilitating access to reliable information, as documented in prior studies information.^10,13,22,23^ Age also emerged as an associated variable: participants aged 25 and older demonstrated significantly higher levels of knowledge compared with those aged 18–24. However, no significant differences were observed among the older age groups (25–29, 30–34, 35–39, and 40–44), suggesting that the primary knowledge gap lies within the youngest cohort, rather than reflecting a gradual increase across all age brackets.^10,13,22,23^ These patterns suggest differential access to fertility education across demographic groups, mirroring disparities observed in international contexts information.^10,16,17^

Other variables related to sexual and reproductive health were also significantly associated with knowledge of the fertile window such as fertility intentions, knowledge, and intention to use FABMs. In relation to fertility intentions, individuals with childbearing experience were more likely to know the fertile window, in contrast to previous systematic reviews that did not find a significant association.^32^

Moreover, knowledge and intention to use FABMs were associated with increased knowledge about the fertile window. These findings highlight the importance of incorporating accurate information about FABMs into educational programs.^19,33^

Finally, it is worth emphasizing that although the item concerning the “most fertile time” is typically used as the primary main indicator of fertile window knowledge,^7,11,16,17^ this study adopts a broader construct composed of four items. This multidimensional measure captures not only the identification of peak fertility but also incorporates awareness of ovulation biomarkers, sperm viability, and correction of common fertility misconceptions. Internal consistency was assessed using Kendall’s Tau-b coefficient, supporting the integration of these items into a single, reliable index.

This broader approach is particularly relevant, as understanding the fertile window encompasses much more than simply identifying the day of peak fertility. It demands comprehensive knowledge of the physiological mechanisms underlying female fertility, such as recognizing ovulation indicators and understanding sperm viability. This need is especially acute in Latin America, where, as previously discussed, significant educational gaps and persistent misinformation about the menstrual cycle and fertility continue to pose challenges.^6,20,24,26^

Despite its contributions, this study has limitations.

Participant recruitment was conducted online and through social media, with the study presented as an anonymous survey on fertility and family planning. Eligible individuals aged 18–44 with Colombian or Ecuadorian nationality were invited to participate voluntarily. However, the use of convenience sampling and online recruitment may have introduced self-selection bias,^34^ as those with a greater interest or knowledge in fertility may have been more likely to participate. Furthermore, the use of self-reported data may introduce bias, as participants could overestimate or underestimate their knowledge, potentially skewing results. Consequently, the sample is not representative of the general population, and the findings should be interpreted with this limitation in mind.

In addition, imbalances in sex and nationality were noted; however, the large sample size enabled robust identification of patterns and sex-based comparisons across Colombian and Ecuadorian contexts.

Finally, fertile window awareness encompasses additional biomarkers—such as basal body temperature, cervical position, and luteinizing hormone levels (detectable *via* home urine tests)—that were not assessed here but merit inclusion in future research.^35^

Moreover, although our study incorporated multiple dimensions of fertile window knowledge and Kendall’s Tau-b coefficient supported the unidimensionality and validity of the proposed index, further psychometric studies are recommended to strengthen its validation and empirical robustness.

Despite these limitations, the findings emphasize the importance of developing targeted educational interventions to enhance understanding of the reproductive cycle and fertility across all demographic groups—including men, individuals of varying socioeconomic backgrounds, and adolescents—to address knowledge disparities and promote equitable reproductive health outcomes.^17,36^ This need is further emphasized by the growing interest in FABMs, which is largely attributed to heightened concerns regarding the adverse effects of hormonal contraceptives.^16,21^

## Conclusions

Knowledge of the fertile window constitutes an essential element of sexual and reproductive health education, influencing informed decision-making in family planning, preconception care, and the prevention of reproductive health challenges.^6^ As the first study to examine this topic specifically in Colombia and Ecuador, this research provides a novel contribution to the regional evidence base. The use of primary data strengthens the reliability of the findings, offering a solid foundation for designing educational strategies tailored to vulnerable populations, including adolescents, men, and individuals of lower socioeconomic status. Results revealed that fertile window knowledge among participants was markedly low, particularly among men and those of lower socioeconomic status. Key sociodemographic predictors included sex, age, socioeconomic status, and educational attainment, while fertility intentions and knowledge of and intent to use FABMs emerged as significant reproductive health correlates. These findings highlight the need for comprehensive fertility awareness education beginning in adolescence. Enhanced understanding of the fertile window could improve fecundability, support preconception health, and reduce fertility knowledge gaps in Latin America.

## Abbreviations Used

FABMs: Fertility awareness-based methods
ILFARUS: Latin American Institute of the Family
STIs: Sexually transmitted infections
